# Ophthalmic manifestation of bilateral ocular toxocariasis: a case-series

**DOI:** 10.1186/s12348-026-00618-6

**Published:** 2026-07-07

**Authors:** Junxiang Gu, Zhujian Wang, Min Zhou, Gezhi Xu, Qian Chen, Qing Chang

**Affiliations:** 1 Department of Ophthalmology, Fudan University Eye and ENT Hospital, Fudan University, 83 Fenyang Rd., Xuhui District, Shanghai, China; 2 Shanghai Key Laboratory of Visual Impairment and Restoration, Fudan University Eye and ENT Hospital, Fudan University, Shanghai, China; 3 NHC Key Laboratory of Myopia and Related Eye Diseases; Key Laboratory of Myopia and Related Eye Diseases, Chinese Academy of Medical Sciences, Shanghai, China; 4 Clinical Laboratory, Fudan University Eye and ENT Hospital, Fudan University, Shanghai, China

**Keywords:** Ocular toxocariasis, Uveitis, Granuloma

## Abstract

**Purpose:**

To explore the clinical features of bilateral ocular toxocariasis (OT).

**Methods:**

In this case-series, the ocular characteristics and treatments of the patients diagnosed with bilateral OT from 2015 to 2024 were reviewed and analyzed. The influences of clinical manifestations on prognosis were evaluated.

**Results:**

Ten patients aged 21 ± 15 years were included. In all the patients, the onset of the two eyes was asynchronous. The first-affected eyes (FAEs) had worse visual acuity than the second-affected eyes (SAEs) (paired t test, *P* = 0.024). Peripheral granulomas were observed in 16 eyes (FAE/SAE: 9/7), followed by posterior granulomas in 3 eyes (all FAEs). The peripheral granulomas in FAEs were longer than those in paired SAEs (6.38 ± 1.69 mm versus 5.29 ± 1.77 mm, *P* = 0.045). Cyclitic membrane, vitreous strand, epiretinal membrane, tractional retinal detachment and ciliary body detachment were observed in 10 (FAE/SAE: 5/5), 6 (FAE/SAE: 3/3), 12 (FAE/SAE: 7/5), 5 (FAE/SAE: 2/3) and 1(FAE) eye at baseline, respectively. The incidences of these lesions didn’t vary greatly from baseline to the latest visits except for an increased incidence of tractional retinal detachment (25% to 60%, *P* = 0.016). Cataract, posterior synechia, band keratopathy and ocular hypertension occurred in 14 (FAE/SAE: 8/6), 9 (FAE/SAE: 6/3), 5 (FAE/SAE: 3/2) and 7 eyes (FAE/SAE: 4/3), respectively. Peripheral granulomas might be correlated with poor visual recovery in this series (Stepwise linear regression, *P* = 0.003).

**Conclusion:**

Bilateral OT cases tended to have an asynchronous onset and asymmetric severity between the two eyes. Such feature could help to maintain the diagnosis of OT and prompt further test of anti-*T. canis* antibody for confirmation despite bilateral involvement.

## Introduction

Ocular toxocariasis (OT) is an uncommon helminthic disease caused by the ocular larval migrans of roundworms *Toxocara canis* or *Toxocara cati*, which mainly affects young patients [1, 2]. OT features nematode endophthalmitis, peripheral granulomas and posterior granulomas, and can further lead to vision-impairing conditions including cataract, retinal folds, macular edema, traction retinal detachment, and even phthisis bulbi [2–4]. Therefore, OT is a notable cause of blindness in children, adolescents and young adults [5, 6].

Patients are infected through unintentional ingestion of embryonated *Toxocara* eggs. The eggs then hatch in the small intestine and the larvae migrate into the mucosa and finally enter the circulation through portal vein system. Rather than migrating to the intestine in young puppies, the larvae in the human body exhibit no tissue tropism and can migrate to various organs including the liver, lung, eye, etc [7, 8]. 

OT typically affects only one eye [2, 9, 10], but bilateral involvement could still be observed in rare cases [5]. The explanation for the extremely low incidence of bilateral OT remains unclear. Besides, the clinical features of bilateral cases haven’t been further explored. In this study, we reviewed the medical records of OT patients with bilateral involvement to seek potential characteristics and disease patterns of bilateral OT.

## Methods

This was a single-centered case series study and was approved by the institutional review board of Eye & ENT Hospital of Fudan University (Approval No. 2020119-1). Written informed consent was waived due to the retrospective design of this study and no invasive examination involved. The study was conducted in accordance with the Declaration of Helsinki.

The medical records of patients diagnosed with bilateral OT at Eye & ENT Hospital of Fudan University from January 2015 to December 2024 were reviewed and evaluated for inclusion according to the criteria similar to our previous study [11]. In brief, bilaterally involved patients were included in the case of (1) typical peripheral or posterior granulomas in at least one eye, or (2) no typical granuloma but vitreous strands or retinal folds with positive anti-*T. canis* antibody outcomes in serum or intraocular fluids, after (3) exclusion of retinoblastoma, Coats disease, persistent fetal vasculature and toxoplasmosis.

Demographic data including sex, age and temporal pattern of bilateral involvement (sequential or simultaneous) were collected. Best correct visual acuity (BCVA) at onset and the latest follow-up visit was recorded. The images of anterior segment photography, fundus photography, retinal optical coherence tomography (OCT), B-scan ultrasonography and ultrasound biomicroscopy (UBM) were evaluated. The medication, complications and surgeries during treatment and follow-up were reviewed.

UBM and B-scan ultrasonography were performed according to the standardized procedure for uveitis of our institution [11, 12]. UBM examinations were performed using BME-300 (MEDA Co., Ltd., China) with a 50 MHz linear imaging system; in addition to regular radial (meridional) scans at 8 principal meridians, attention was paid to the pars plana, peripheral vitreous and peripheral retina. For the peripheral granulomas detected through UBM, both radial and transverse (coronal) sections were captured; the location, longest diameter and thickness of each granuloma were recorded; the size of the largest granuloma was selected for statistics when multiple granulomas were observed. B-scan ultrasonography was performed using the Aviso ophthalmic ultrasound platform (Quantel Medical, France) at 10 MHz; swept transverse scans in the 4 cardinal directions and axial scans were conducted to evaluate peripheral and posterior lesions, respectively. OCT images were captured using Spectralis HRA + OCT (Heidelberg Engineering, Germany). Anti-*T. canis* antibody levels were detected by an enzyme-linked immunosorbent assay (ELISA) kit (RE58721; IBL International, Inc., Germany). The antibody level unit (U) was calculated as (sample absorbance×10)/cut-off according to the manual of the kit; antibody levels in serum samples higher than 11U were considered positive [13].

Normality was evaluated by the Shapiro-Wilk test. Continuous variables distributed normally were presented in the form of mean ± standard deviation; non-normally distributed variables were presented as median (interquartile range). BCVA measurements were transformed to logarithm of the minimum angle of resolution (logMAR). In order to seek potential intra-individual differences between the two eye of each patient, paired comparisons were performed; BCVA and sizes of granulomas were compared using paired t tests; numbers of granulomas were compared using Wilcoxon signed ranks tests; presence of lesions or complications were compared using McNemar tests. Similarly, in order to analyze the longitudinal trends within each eye, corresponding statistical tests were applied to the paired data at baseline and the latest follow-up visit of each eye. Stepwise linear regression was performed to explore potential factors for visual prognosis; latest BCVA measurements or differences between baseline BCVA and latest BCVA were selected as the dependent variables, while laterality, age, baseline BCVA, characters of granulomas, inflammatory lesions, complications and treatments were selected as independent variables in the regression. The statistical analysis was performed using SPSS software (version 22.0; SPSS Inc., USA). P values lower than 0.05 were considered statistically significant.

## Results

### Baseline characteristics

Between 2015 and 2024, 10 patients diagnosed with OT were confirmed to have bilateral involvement and included in the analyses. Among them, 6 were male and 4 were female. Nine patients were diagnosed via the observation of typical granulomas; the remaining one patient presented with bilateral vitritis without granulomatous lesion, and was then diagnosed through positive serum anti-*T. canis* antibody (18.6 U). The mean age at the first visit was 21 ± 15 (95% confidence interval [95%CI] 10–32) years. The mean duration of follow-up was 39.7 (95%CI 11.0–68.4) months.

All patients had a sequential onset of OT. The condition occurred firstly in the left eye of 7 patients, while the right eye was initially affected in the other 3 patients. No clinically evident symptoms suggestive of visceral larva migrans (VLM) were documented. The mean baseline BCVA (in logMAR) was 1.59 ± 0.90 (95%CI 0.65–2.53) for the first-affected eyes (FAEs) and 0.55 ± 0.31 (95%CI 0.22–0.87) for the second-affected eyes (SAEs). The BCVA of FAEs was worse than that of SAEs (paired t test; *P* = 0.024).

The details were listed in Table 1.

**Table 1:** Baseline characteristics of the 10 patients with bilateral OT

Characteristic	Value
**Age** (mean ± SD, years)	21 ± 15
**Sex** (female/male)	
Male	6
Female	4
**Diagnostic approach**	
Bilateral granuloma	7
Unilateral granuloma	2
No granuloma but positive antibody in serum	1
**First-affected eye** (right/left)	
Right	3
Left	7
**BCVA in LogMAR** (mean ± SD)	
First-affected eye	1.59 ± 0.90
Second-affected eye	0.55 ± 0.31

Abbreviation: SD, standard deviation; BCVA, best correct visual acuity; LogMAR, logarithm of the minimum angle of resolution

### Clinical manifestations

#### Granulomas

**Fig. 1:**
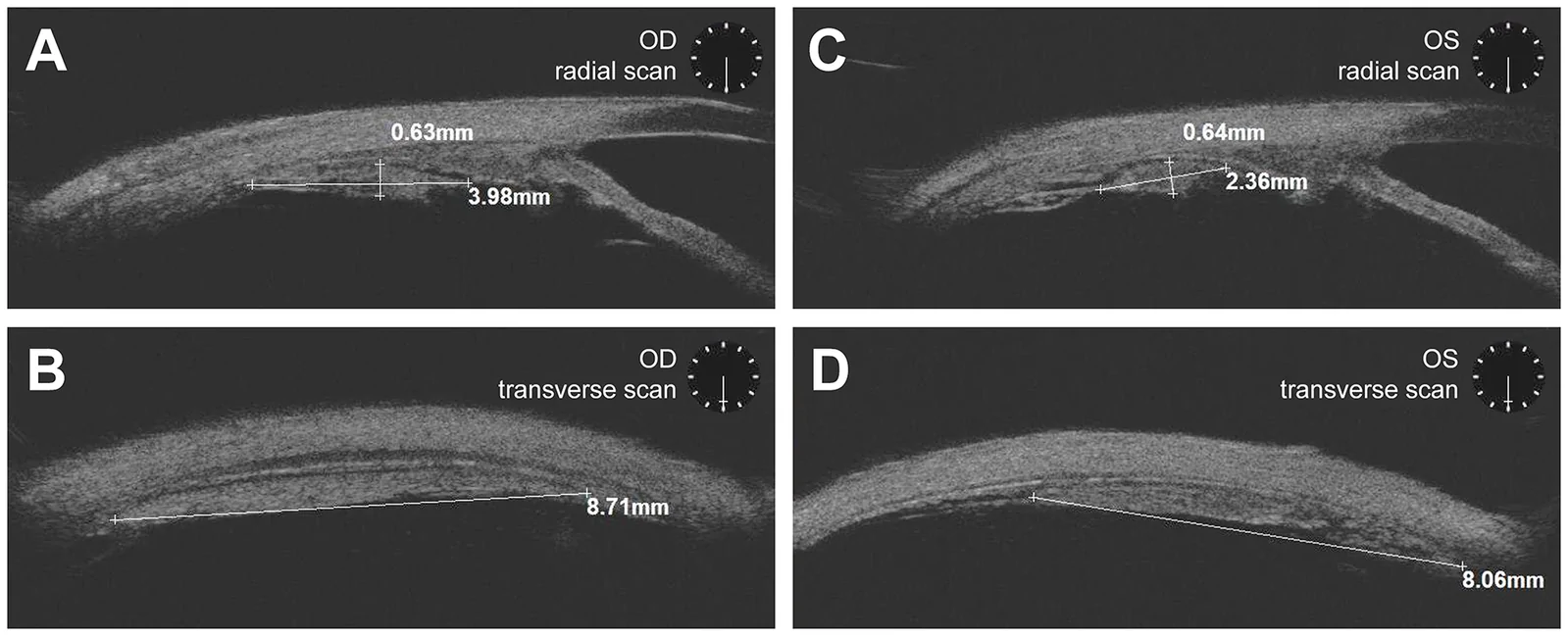
A typical peripheral granuloma on UBM. The figure shows typical peripheral granulomas present bilaterally in one patient, as detected via radial (meridional) scans (**A** and **C**) and transverse (coronal) scans (**B** and **D**) of UBM at the 6 o’clock position of the pars plana. The granuloma appears as a well-defined, hyperechoic masses whose long axe is aligned tangentially to the ciliary body on the transverse scan and short axe is approximately perpendicular to the ocular wall. Laterality, location and orientation of the scans are indicated in the top-right corner of each panel. Abbreviation: OD, oculus dexter (right eye); OS, oculus sinister (left eye)

**Fig. 2:**
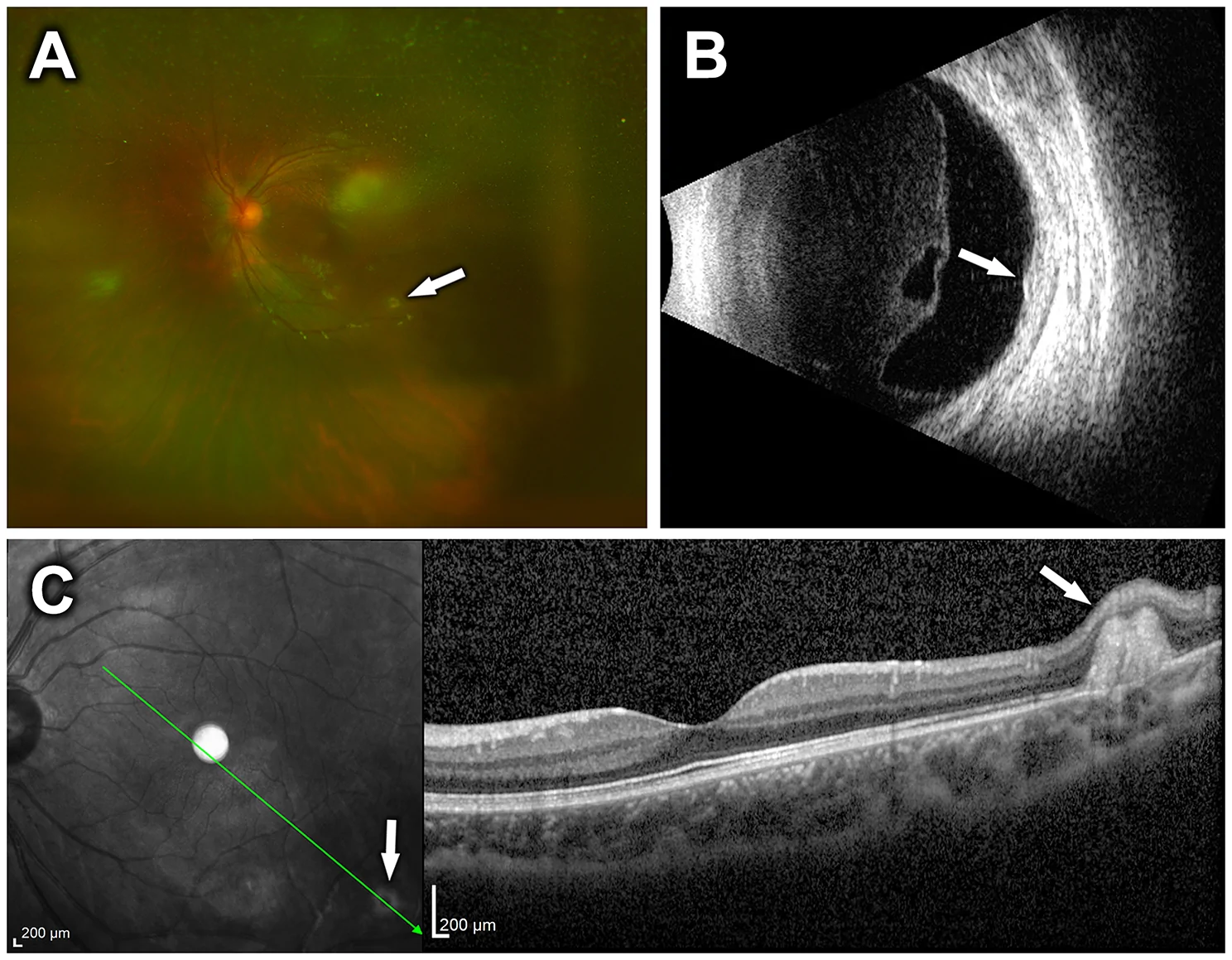
Multimodal imaging of a posterior granuloma. This figure shows the same posterior granuloma visualized through ultra-widefield fundoscopy (**A**), B-scan ultrasonography (**B**) and OCT (**C**). **A**: The granuloma appears as a yellowish mass (arrow) located in the deep retina on fundoscopy. **B**: On B-scan ultrasonography, the granuloma appears as a focal thickening (arrow) of the retina with moderate-to-high echogenicity. **C**: The infrared image on the left shows the location of the granuloma (arrow) and the scanning line (green arrow-headed line) of OCT. The cross-sectional image of the OCT scan shows that the granuloma (arrow) locates in the outer layer of the retina

Peripheral granulomas were observed using UBM and appeared as well-defined, hyperechoic masses configured as triaxial ellipsoids whose long axes (length) were consistently detected aligning tangentially to the ciliary body on transverse (coronal) scans and short axes (thickness) were approximately perpendicular to the ocular wall (Fig. 1). Posterior granulomas were evaluated via the combination of fundus photography, OCT and B-scan ultrasonography, which were identified as hyperintense subretinal lesions (Fig. 2).

In the FAEs, 6 eyes (60%) had peripheral granulomas only; 3 eyes (30%) presented with combined anterior and posterior granulomas. While in the SAEs, peripheral granulomas were detected in 7 eyes (70%); no posterior granuloma was observed (Table 2). As for the number of granulomas in each of the eye, 4 eyes (20%) had no granuloma, 10 eyes (50%) had one granuloma, and 6 eyes (30%) had two granulomas; the median numbers were 1 (range 1–2) and 1 (range 1–2) for the FAEs and SAEs, respectively, which yielded no statistical significance (Wilcoxon signed ranks test; *P* = 0.359).

**Table 2:** Clinical manifestations and complications of the eyes

Clinical parameter	Laterality	
	FAE (*N* = 10 eyes)	SAE (*N* = 10 eyes)
**Type of granulomas**	*Number of eyes*	
Peripheral	6	7
Posterior	0	0
Combined (peripheral and posterior)	3	0
No granuloma	1	3
**Distribution of peripheral granulomas**	*Number of granulomas*	
Inferior	3	6
Inferonasal	3	2
Inferotemporal	3	1
Superonasal	1	0
**Inflammatory lesions (at baseline and [latest visits])**	*Number of eyes*	
Cyclitic member	5 [7]	5 [7]
Vitreous strands	3 [5]	3 [6]
Epiretinal membrane	7 [8]	5 [7]
Tractional retinal detachment	2 [6]	3 [6]
Ciliary body detachment	1 [2]	0 [0]
**Anterior segment complications**	*Number of eyes*	
Cataract	8	6
Posterior synechia	6	3
Band keratopathy	3	2
Ocular hypertension	4	3

Abbreviation: BCVA, best correct visual acuity; logMAR, logarithm of the minimum angle of resolution; SD, standard deviation; FAE, first-affected eye; SAE, second-affected eye

Nineteen peripheral granulomas were observed in 16 eyes. Eighteen peripheral granulomas were detected on the surface of the ciliary body and 1 granuloma was detected on the surface of the peripheral retina. Among these peripheral granulomas, 9 granulomas were located inferiorly, 5 inferonasally, 4 inferotemporally, and 1 superonasally (see Table 2). The peripheral granulomas of the FAEs had a mean length (longest diameter) of 6.38 ± 1.69 (95%CI 4.61–8.16) mm and a mean thickness of 0.82 ± 0.27 (0.53–1.10) mm; those of the SAEs had a mean length of 5.29 ± 1.77 (95%CI 3.73–7.14) mm and a mean thickness of 0.84 ± 0.35 (0.48–1.21) mm. The granulomas in FAEs had greater lengths than in paired SAEs (paired t test; *P* = 0.045); no difference was observed in thickness between granulomas of paired eyes (paired t test; *P* = 0.831).

#### Other inflammatory lesions

**Fig. 3:**
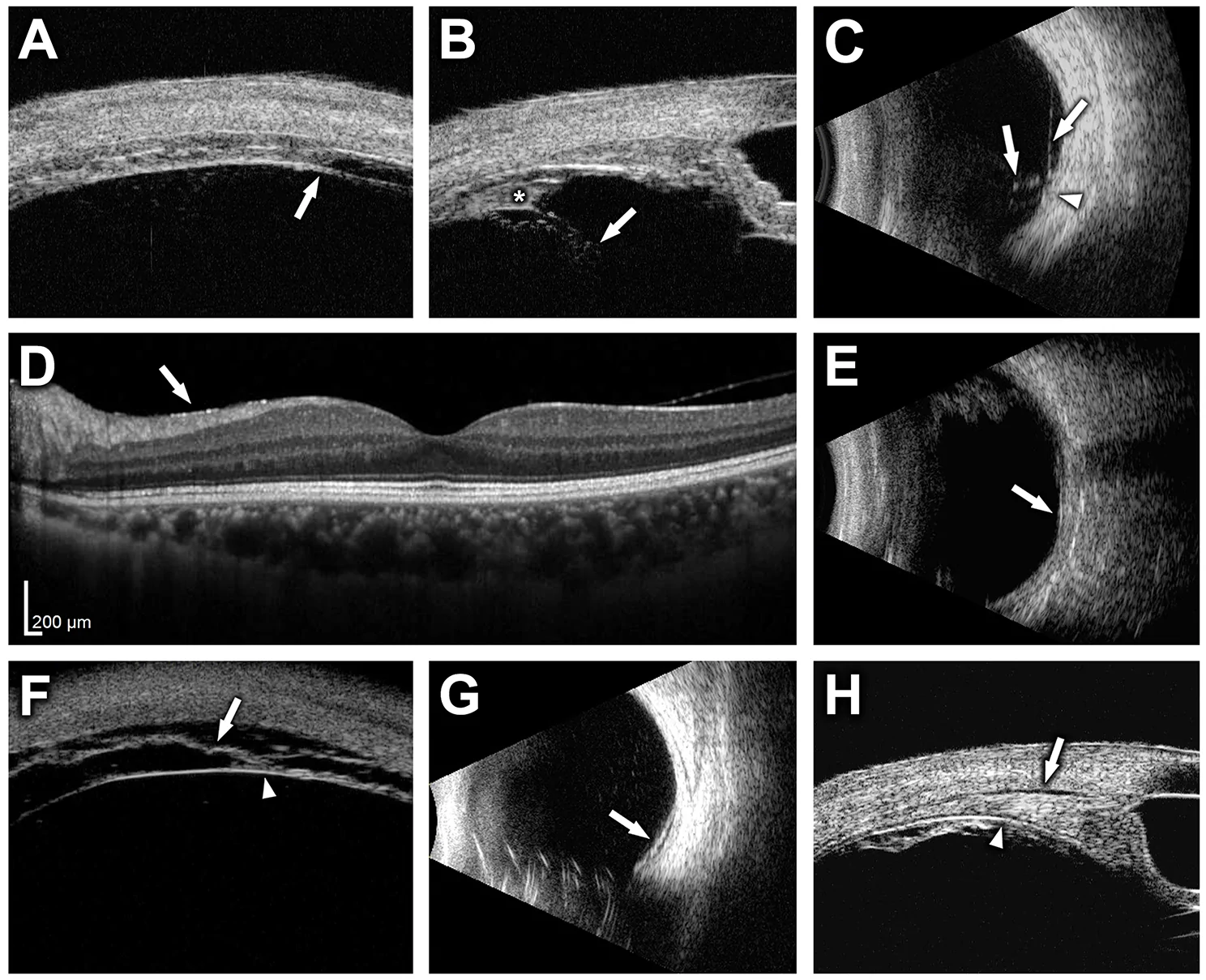
Other lesions caused by inflammation and proliferation. Other lesions observed included cyclitic membrane (**A**), vitreous strand (**B-C**), epiretinal membrane (**D-E**), tractional retinal detachment (**F-G**) and ciliary body detachment (**H**). **A**: A transverse (coronal) UBM scan revealed a hyperechoic cyclitic membrane (arrow) on the surface of the ciliary body. **B**: Another radial (meridional) UBM scan demonstrated a vitreous strand (arrow) extending from a peripheral granuloma (asterisk) into the vitreous cavity. **C**: Vitreous strands (arrows) detected by an anteroposterior paraxial scan of ultrasonography extended from a posterior granuloma (arrowhead). **D-E**: Epiretinal membrane (arrows) could be visualized by either OCT (**D**) or B-scan ultrasonography (**E**). **F**: A transverse scan of UBM showed a detachment of the peripheral retina (arrow) caused by traction of an adhered proliferative membrane (arrowhead). **G**: A transverse scan of B-scan ultrasonography showed a detachment of the inferotemporal peripheral retina (arrow). **H**: A radial scan of UBM suggested a detachment of the ciliary body (arrow) possibly caused by the traction of a cyclitic membrane (arrowhead)

Other lesions caused by inflammation or proliferation included cyclitic membrane, vitreous strand, epiretinal membrane, tractional retinal detachment and ciliary body detachment (Fig. 3), which at baseline occurred in 5 (50%), 3 (30%), 7 (70%), 2 (20%) and 1 (10%) eye among the FAEs, respectively, compared with 5 (50%), 3 (30%), 5 (50%), 3 (30%) and 0 (0%) eye among the SAEs; at the latest follow-up, the frequencies of these lesions were 7 (70%), 5 (50%), 8 (80%), 6 (60%) and 2 (20%) eyes among the FAEs, respectively, compared with 7 (70%), 6 (60%), 7 (70%), 6 (60%) and 0 (0%) eye among the SAEs (Table 2). The incidences of cyclitic membrane, vitreous strand, epiretinal membrane and tractional retinal detachment between the paired eyes were of no significant difference at neither baseline (McNemar test; *P* > 0.999, *P* > 0.999, *P* = 0.625 and *P* > 0.999, respectively) or the latest follow-up (McNemar test; all *P* > 0.999); the incidence of ciliary body detachment was not comparable due to its absence in SAEs. Longitudinally, the incidence of tractional retinal detachment rise significantly at the latest follow-up when compared to baseline (McNemar test; *P* = 0.016); detachment involving the posterior retina was observed in one eye (FAE) throughout the study. The incidences of cyclitic membrane, vitreous strand, epiretinal membrane and ciliary body detachment didn’t change greatly from the baseline to the latest follow-up (McNemar test; *P* = 0.250, *P* = 0.125, *P* = 0.375 and *P* > 0.999, respectively).

### Anterior segment complications

Cataract occurred in 14 eyes of 8 patients (8 FAEs and 6 SAEs); posterior synechia occurred in 9 eyes of 9 patients (6 FAEs and 3 SAEs); band keratopathy occurred in 5 eyes of 3 patients (3 FAEs and 2 SAEs); ocular hypertension occurred in 7 eyes of 6 patients (4 FAEs and 3 SAEs) (see Table 2). The differences in the frequencies of these complications between paired FAEs and SAEs were not significant (McNemar test; *P* = 0.500, *P* = 0.250, *P* = 0.500 and *P* > 0.999, respectively).

### Treatment

The treatments were detailed in Table 3.

**Table 3:** Treatments received by the patients

Patient	Corticosteroid	Immunomodulatory agents	Anthelmintics	Surgery	Ocular hypotensive agent
1	Topical	MTX & Adalimumab	/	OD (SAE): cataract OS (FAE): cataract & PKP	/
2	Systemic	/	/	/	/
3	Topical	/	/	/	/
4	/	/	/	OD (FAE): LPI	OD (FAE): carteolol hydrochloride & brimonidine tartrate
5	Topical & systemic	MTX	/	OD (SAE): cataract & PI OS (FAE): cataract	OD (SAE): brinzolamide & timolol & latanoprost
6	Topical & systemic	MTX & Tofacitinib & Adalimumab	Albendazole*	/	OU: brinzolamide
7	Topical	/	/	/	/
8	Topical & systemic	/	Albendazole*	OS (FAE): cataract	OD (SAE): carteolol hydrochloride
9	Topical & systemic	/	/	OS (SAE): LPI	OD (FAE): brinzolamide & timolol
10	Topical	/	/	OS (FAE): cataract	OS (FAE): carteolol hydrochloride

* Albendazole (400 mg twice daily for 2 weeks) was administered to patients weighing ≥ 60 kg, under the coverage of systemic corticosteroids

Abbreviation: MTX, methotrexate; PKP, penetrating keratoplasty; LPI, laser peripheral iridotomy; PI, peripheral iridotomy; PPV, pars plana vitrectomy; FAE, first-affected eye; SAE, second-affected eye; OD, oculus dexter (right eye); OS, oculus sinister (left eye); OU, oculus uterque (both eyes)

Four patients received combined systemic and topical corticosteroid therapy; 4 patients received topical corticosteroid therapy only; 1 patient received systemic corticosteroid therapy only; 1 patient didn’t receive any administration of corticosteroid.

Antiparasitic treatment was not administered to eight patients with no active disease during follow-up. Two patients (Patient 6 and 8) who experienced flares, both weighing ≥ 60 kg, were advised to receive albendazole (400 mg twice daily for 2 weeks) with concurrent systemic corticosteroids and were referred to infectious disease specialists at a general hospital for continued antiparasitic management. Ophthalmic follow-up at our institution was discontinued after referral, and post-referral data were not included in the present analysis.

Three patients had been initiated on immunomodulatory agents by immunologists prior to referral. Patient 1 received methotrexate (MTX) and adalimumab, and Patient 5 received MTX; these agents were prescribed for severe anterior segment complications (cataract and band keratopathy in Patient 1; cataract in Patient 5). After surgical management of these anterior segment complications (detailed below) and a period of relapse-free observation at our institution, we recommended tapering these medications under the immunologists’ guidance. Patient 6, previously diagnosed with juvenile idiopathic arthritis (JIA; previous medical records were not available), had received systemic corticosteroids, MTX, tofacitinib, and adalimumab owing to frequent relapses. Upon referral, we identified coexistent OT; after a relapse during follow‑up, albendazole therapy was then initiated at a general hospital. At the latest telephone follow-up, no signs of relapse were observed since the completion of antiparasitic therapy, and the patient was returned to the immunologist, who continued tofacitinib and MTX for JIA control.

Six eyes of 4 patients underwent cataract surgery, among which one eye received combined penetrating keratoplasty due to corneal endothelial decompensation. Given the posterior retinal detachment in one eye already presenting with phthisis bulbi and light perception vision, vitrectomy was not performed due to limited anticipated benefit. Three eyes of 3 patients received peripheral iridectomy; 7 eyes of 6 patients were treated with topical ocular hypotensive medication. The intraocular pressure (IOP) of all these eyes was controlled below 21 mmHg.

### Visual outcome and prognosis

At the latest follow-up visit, the mean BCVA was 1.13 ± 1.25 (95%CI -0.18–2.44) for the FAEs and 0.30 ± 0.35 (95%CI -0.07–0.66) for the SAEs. No significant difference in BCVA was observed between paired FAEs and SAEs (paired t test; *P* = 0.134).

Stepwise linear regression was performed to investigate factors possibly affecting visual prognosis. Independent variables analyzed included laterality (FAE or SAE), age, baseline BCVA, peripheral granulomas, posterior granulomas, number of granulomas, cyclitic membrane, vitreous strand, epiretinal membrane, tractional retinal detachment, ciliary body detachment, cataract, posterior synechia, ocular hypertension, band keratopathy, cataract surgeries and administration of corticosteroid, immunosuppressants and biologics. The variables entered the regression were baseline BCVA (B = 1.003, *P* < 0.001) and presence of peripheral granulomas (B = 1.270, *P* = 0.006); the P value of the regression was < 0.001.

We defined another parameter, vision difference, as the BCVA at the latest follow-up minus the baseline BCVA to represent the change in visual outcomes, and attempted to seek potential factors of it through another stepwise linear regression. The independent variables were the same except for baseline BCVA because it had already been included in the calculation of vision difference. The variable entered the regression was presence of peripheral granulomas (B = 1.269, *P* = 0.003); the P value of the regression was 0.003.

The findings above indicated that visual function at baseline was positively correlated with visual prognosis, and that the presence of peripheral granulomas might be correlated to poor visual prognosis and recovery.

## Discussion

In this study, we reviewed manifestations and prognosis of bilateral OT cases, and found that the two eyes of each patient had asynchronous onset and dissimilar severity. Such findings might help to explain the pathogenesis and rarity of bilateral OT.

Although OT is mainly unilateral [2, 9, 14], several studies showed that bilateral involvement was observed in rare cases [5, 15]. Therefore, a bilateral condition could not preclude the diagnosis of OT. The incidence of bilateral cases remains unclear. Stewart et al. reported 2 bilateral cases among 22 patients with OT [5]. In order to estimate the proportion of bilateral cases among all OT patients, we conducted a search for cases diagnosed with OT during the same period (2015–2024), which yielded 178 records. Thus, the estimated percentage of bilateral OT was 5.6% (95%CI 2.8–9.6%) at our institution during this period, which was comparable to the ratio (2/22) in the study of Stewart et al. [5]

The underlying explanation for the rarity of bilateral OT remains unknow. Similarly, other parasitic diseases also demonstrate predominantly unilateral ocular involvement [16, 17]. One possible hypothesis is that the migrations of larvae into the two eyes are independent events because the blood supply of the two eyes has no bilateral anastomosis and branches at a proximal location. The blood supply of the right eye originates from the brachiocephalic trunk (innominate artery) while that of the left eye originates from the left common carotid artery, which makes it hard for a single batch of larvae to diverge and enter these two arteries at same time due to the rapid blood flow at the aorta arch. Therefore, the larvae in the two eyes of bilateral OT cases might come from different batches or waves, and the bilateral involvement might require the very small joint probability of larvae entering both two eyes independently and successively. Supporting this hypothesis are the facts that the patients in this study had asynchronous onsets in the two eyes, and that the proportion of bilateral cases over all OT cases in our institution (5.6%) was comparable to the estimated overall prevalence of 5% (95%CI: 3% − 6%) derived from a meta-analysis [18].

Another feature of bilateral OT was the asymmetric severity. In our study, the FAEs tended to have larger granulomas and lower baseline BCVA when compared to its paired SAEs. Such findings were consistent with the different interocular presentation in a case reported by Lee et al. [15] One possible explanation of the asymmetry in severity is that the asynchronous onsets of the two eyes result in different durations of inflammation and granuloma formation; thus, the longer period of inflammatory damage in the FAE leads to worse outcomes. Moreover, the disease of the SAE could be detected at an earlier stage due to enhanced patient education, disease awareness and ophthalmic follow-up, which further added to the difference in severity between the two eyes. Besides, by analogy with the studies on other parasitic diseases [19, 20], we infer that the longer course of inflammation in the FAE might induce the establishment of immune tolerance which could relieve the severity in the fellow eye. Another possibility, though not applicable to this study (all the patients in this study received systemic treatment after bilateral onset), is that systemic treatment for the FAE could attenuate the manifestations of the SAE, thus contributing to the asymmetry in severity.

Our analysis on the visual prognosis showed that latest BCVA was positively correlated with baseline BCVA, which was consistent with the study by Zhang et al. [21] Besides, the presence of peripheral granulomas was correlated with less amount of BCVA improved in this study, while the study by Kwon et al. suggested that unfavorable visual outcomes were associated with posterior granulomas [22]. A possible reason is that the patients in our study had a higher rate of vision-threatening anterior segment complications including posterior synechia, band keratopathy and high IOP [3], which might be the consequences of peripheral granulomas.

In this case series, three patients had been initiated on immunomodulatory agents before referral. For Patients 1 and 5, MTX with or without adalimumab was prescribed for severe anterior segment complications, consistent with previous reports in pediatric uveitis [23, 24]. Patient 6, who had JIA, received MTX and adalimumab for JIA‑associated uveitis [25], and tofacitinib was added owing to disease flares [26]. Our subsequent management focused on achieving surgical stability and a relapse‑free observation period, after which we recommended tapering these medications under the referring specialists’ guidance.

Several limitations of this study should be acknowledged. First, given the rarity of OT cases (especially bilateral ones), the sample size is relatively small, which may limit the power of the statistical analyses. To mitigate this constraint, paired tests were employed to minimize the effects of inter-individual variation. Second, the retrospective design inherently carries risks of incomplete data. In particular, the interval between the onsets of the two eyes in most cases was neither recorded nor accurately recalled during follow-up visits, which restricted our further exploration of the pattern of bilateral involvement. Third, anti‑T. canis antibody detection was not a readily available examination in the early years of the study period. In the absence of serological confirmation, cases without typical granulomas might have been underdiagnosed; bilateral cases were even less likely to be considered for OT. Therefore, the data in our study may underestimate the true number of OT cases. Additionally, a potential selection bias should be acknowledged. Bilateral OT may carry an increased risk of concurrent VLM, but no systemic symptoms were recorded in our cohort, likely because they were self‑limiting, misattributed to common childhood infections, or filtered out by referral to a tertiary ophthalmic center. Our findings therefore primarily apply to patients with chronic disease in the post‑acute phase.

## Conclusions

The involvement of the second eye could be an independent event and might not necessarily exclude the diagnosis of OT; in such circumstances, antibody detection could aid in the diagnosis of bilateral OT. The onsets in the two eyes are usually asynchronous, leading to the greater severity and poorer vision in the first affected eye. The presence of peripheral granulomas could also be one of the negative factors affecting visual prognosis and recovery in OT patients.

## Data Availability

No datasets were involved in this study. Additional data analyzed during the current study are available from the corresponding author on reasonable request.

## Abbreviations

OT: Ocular toxocariasis
FAE: First-affected eye
SAE: Second-affected eye
BCVA: Best correct visual acuity
OCT: Optical coherence tomography
UBM: Ultrasound biomicroscopy
ELISA: Enzyme-linked immunosorbent assay
logMAR: Logarithm of the minimum angle of resolution
IOP: Intraocular pressure
